# Statistical Correlations between HPLC Activity-Based Profiling Results and NMR/MS Microfraction Data to Deconvolute Bioactive Compounds in Mixtures

**DOI:** 10.3390/molecules21030259

**Published:** 2016-02-24

**Authors:** Samuel Bertrand, Antonio Azzollini, Andreas Nievergelt, Julien Boccard, Serge Rudaz, Muriel Cuendet, Jean-Luc Wolfender

**Affiliations:** 1 School of Pharmaceutical Sciences, University of Geneva, University of Lausanne, Quai Ernest-Ansermet 30, CH-1211 Geneva 4, Switzerland; Antonio.Azzollini@unige.ch (A.A.); andreas.nievergelt@gmail.com (A.N.); Julien.Boccard@unige.ch (J.B.); Serge.Rudaz@unige.ch (S.R.); Muriel.Cuendet@unige.ch (M.C.); Jean-Luc.Wolfender@unige.ch (J.-L.W.); 2 Groupe Mer, Molécules, Santé-EA 2160, UFR des Sciences Pharmaceutiques et Biologiques, Université de Nantes, 9 Rue Bias, BP 53508, F-44035 Nantes, France

**Keywords:** microfractionation, biological profiling, correlation analysis, quinone reductase induction, metabolomics

## Abstract

Recent approaches in natural product (NP) research are leading toward the discovery of bioactive chemical entities at the microgram level. In comparison to classical large scale bioassay-guided fractionation, the use of LC-MS metabolite profiling in combination with microfractionation for both bioactivity profiling and NMR analysis, allows the identification of bioactive compounds at a very early stage. In that context, this study aims to assess the potential of statistic correlation analysis to enable unambiguous identification of features related to bioactive compounds in mixtures, without the need for complete isolation. For that purpose, a mixture of NPs was microfractionated by rapid small-scale semi-preparative HPLC for proof-of-concept. UHPLC-ESI-TOFMS profiles, micro-flow CapNMR spectra and a cancer chemopreventive assay carried out on every microfraction were analysed by statistical correlations.

## 1. Introduction

Natural products (NPs) are still an important source of novel bioactive compounds for pharmaceutical companies, even though the latter have ceased or significantly reduced their NP drug discovery programs [1,2]. Notwithstanding Nature provides a massive reservoir of organisms able to produce potentially beneficial compounds to be discovered and explored [3], several reasons can explain this trend [4] and some of them are specifically related to NP drug discovery such as: (1) difficulties in sourcing/harvesting samples [4]; (2) the lack of compatibility between traditional bioactivity guided fractionation with the fast cycle of high-throughput screening campaigns [5,6]; (3) the constant rediscovery of known NPs [7,8]. These latter two factors emphasize the need for the development of innovative strategies allowing the rapid assessment of bioactive molecules within a crude plant extract, for the fast purification and identification of compounds of interest [9,10].

In recent years, new strategies were developed to drastically speed up the detection and localisation of bioactive compounds in crude extracts. One approach consists in the development of high resolution biological profiling based on high performance liquid chromatography (HPLC), which enables highlighting bioactive constituents of a crude extract using only milligram quantities [11,12]. This approach takes advantage of the high-resolution capabilities of small-scale semi-preparative HPLC to separate all the constituents of a crude extract with the possibility to evaluate their bioactivity already with minute amounts of injected extracts. According to the type of bioassay, two different strategies are predominantly used: (1) the direct on-line coupling of HPLC with bioassays [12,13] (such as for antioxidant properties [14], acetylcholine esterase [15] or cathepsin B [16] inhibition) and (2) at-line post chromatographic bioactivity evaluation after microfractionation of an extract, which involves drying and re-dissolving the fractions in a suitable biocompatible medium. The latter strategy has largely been employed for simple chemical assays (such as antioxidant activity [17]), enzymatic assays and even for cell-based bioassays [11,12,18]. *In vivo* assays could also be performed directly at-line with miniaturised setups, particularly those involving zebrafish [19,20].

This high-resolution biological profiling approach allows the localisation of LC peaks responsible for the activity in the chromatogram. In addition, structural information may be generated on-line or at-line from the LC peak permitting identification of the compounds responsible for the activity by accurate chemical analysis using mass spectrometry (MS) or miniaturised nuclear magnetic resonance (NMR) methods combined with database search (*i.e.*, dereplication [18,21,22,23,24,25]). Dereplication, combined with high resolution biological profiling, thus represents a rational and efficient method for the early identification of bioactive compounds [26]. If quantitative information can be retrieved from microfractionation experiments, IC_50_ values can furthermore be determined at this stage [19]. Unfortunately, the identification of bioactive compounds within a biologically active extract is not as trivial as it seems. Sometimes, the observed activity is linked to a chromatographic zone where multiple compounds are coeluting; in that particular case, the selection of the active constituent remains difficult [27].

Some strategies combining both types of experiments (LC-MS and NMR) called Statistical HeterospectroscopY (SHY) take advantage of both data sources to highlight signals from the ^1^H-NMR acquisition related to a LC-MS feature [28,29,30]. This is generally performed between the ^1^H-NMR and LC-MS profiles of crude mixtures coming from different situations. Thus, spectroscopic information of MS features are highlighted without the need of physical separation. They correspond to NMR signals exhibiting positive correlations with specific MS features. A similar approach is proposed in the present study to overcome coelution problems. Statistical correlations between LC-MS, NMR and/or bioactivity data were evaluated for their help in the deconvolution of profiles obtained from microfractions. This should eventually help a more accurate identification of the active principles in a crude extract by rapid partial fractionation without the need for complete purification [31].

For that purpose, a mixture of 18 compounds, including a compound known to be bioactive, was microfractionated using semi-preparative HPLC. Each fraction was further profiled by high-throughput ultra-high pressure liquid chromatography (UHPLC) coupled to high-resolution (HR) MS and micro-NMR. Ultimately, they were evaluated in a cell-based assay for quinone reductase (QR) induction activity. Statistical correlations [32,33,34] between LC-MS features, NMR features and/or QR induction recorded for each microfraction was used to deconvolute coeluting compounds and unambiguously highlight features of the bioactive compound.

## 2. Results and Discussion

An artificial extract (AE) consisting of 18 compounds was prepared. They all displayed various physicochemical properties and responded differently to UV and MS detection. The chromatographic separation of the AE constituents was optimised at the analytical scale and further geometrically transferred to the semi-preparative scale [35,36]. To be generically applicable to 96 well plates based bioassays, fractionation was restricted to 80 fractions (16 wells were kept for controls) with a maximum volume of 1.7 mL for direct evaporation in deep-well plates without cross-contamination [26]. To fulfil these requirements, a column of 250 × 10 mm was chosen and the HPLC gradient time was limited to 28 min at a flow-rate of 5 mL/min. The scale of the chosen microfractionation and column geometry is compatible with a loading of tens of milligrams of mixtures. This setup led to microfractions containing sufficient amounts of constituents for both HPLC-based activity profiling and further LC-MS and NMR analysis [26,27]. This format has already been reported to be compatible with dereplication by either MS or NMR [26].

**Figure 1:**
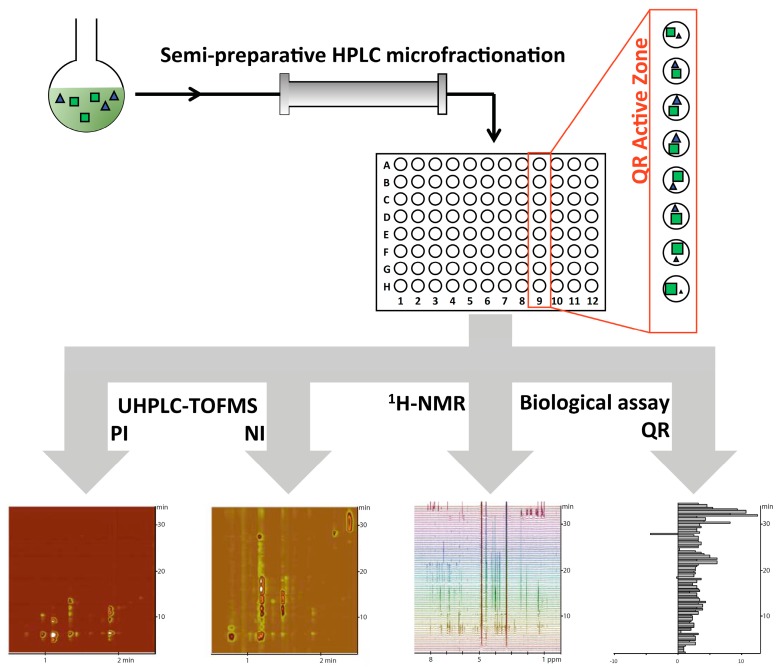
General strategy to evaluate statistical correlations for deconvolution of spectral signals. The 18-compounds mixture was microfractionated by semi-preparative reversed phase HPLC and each fraction was profiled using UHPLC-TOFMS in positive (PI) and negative (NI) ionization modes, and ^1^H-NMR. In addition, each well was evaluated in a cell-based assay for quinone reductase (QR) induction activity.

The AE was thus submitted to small-scale semi-preparative microfractionation by reversed phase HPLC. This yielded 80 microfractions that were transferred to a deep-well microtiter plate (Figure 1). All microfractions were profiled using UHPLC-TOFMS in positive and negative ionization (PI and NI) modes, and ^1^H-NMR. On one side, all *m*/*z* features of the microfraction LC-MS fingerprints were automatically extracted using MZmine 2 software [37] and two data matrices, one for each ionisation mode, were generated. On the other side, the ^1^H-NMR profiles were bucketed to generate a complementary orthogonal data matrix. In this case residual methanol and water signals were removed.

### 2.1. Microfractionation Combined with Statistical HeterospectroscopY to Improve Compound Identification

To deconvolute the NMR signals of compounds not completely separated by chromatography, as often occurs in natural extracts, Statistical HeterospectroscopY (SHY) was used. This method takes advantage of both data sources, highlighting signal from the ^1^H-NMR acquisition related to LC-MS features [28,29,30,32,33]. In our case, SHY MS-NMR correlations were established between features coming from both ^1^H-NMR and LC-MS profiles for each microfraction [32,33]. In the case of LC-peak coelution, the ratio of constituents eluting slightly differently, within microfractions should permit their deconvolution without the need for a fully resolved chromatographic separation.

**Figure 2:**
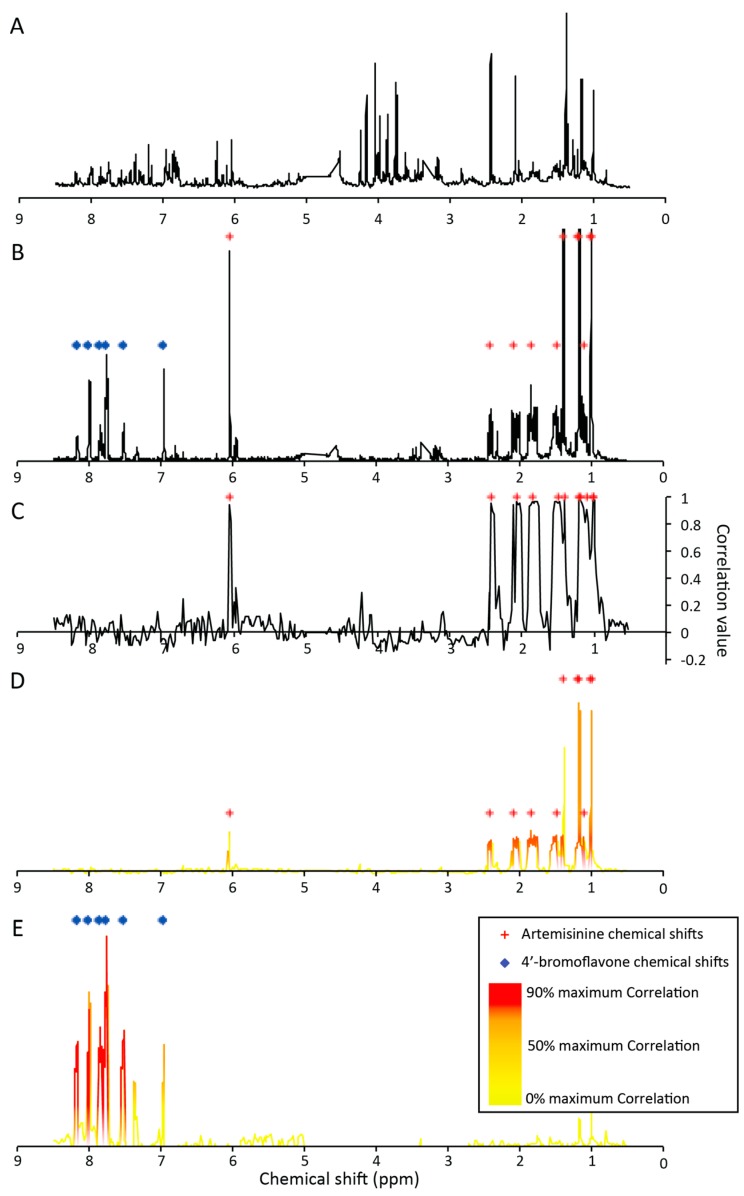
Example of statistical deconvolution on the coelution of artemisinin and 4′-bromoflavone. (**A**) sum of all 80 ^1^H-NMR spectra; (**B**) sum of all 80 ^1^H-NMR spectra of fractions G9 to H10; (**C**) correlation ^1^H-NMR pseudospectrum, corresponding to feature RT = 2.18 min and *m*/*z* = 283.1566 (PI)—artemisinin as [M + H]^+^ adduct; (**D**) filtered ^1^H-NMR pseudospectrum corresponding to feature RT = 2.18 min and *m*/*z* = 283.1566 (PI)—artemisinin as [M + H]^+^ adduct; (**E**) filtered ^1^H-NMR pseudospectrum corresponding to feature RT = 2.37 min and *m*/*z* = 300.9882 (PI)—4′-bromoflavone as [M + H]^+^ adduct; red crosses (+) highlight chemical shifts detected for pure artemisinin; blue diamonds (♦) highlight chemical shifts detected for pure 4′-bromoflavone.

In the presented dataset, features observed in LC-MS were thus correlated with ^1^H-NMR buckets to highlight chemical shifts related to each specific MS feature. This approach was applied to generate “pure” ^1^H-NMR pseudo-spectra for MS peaks in the mixture coeluting under the conditions used for activity-based profiling. As an example the UHPLC-TOFMS analyses of the microfractions revealed two intense ions detected in 11 consecutive fractions (G9 to H10) indicating coelution. These MS features were recorded in the PI mode (*m*/*z* = 283.1566 at RT = 2.18 min and *m*/*z* = 300.9882 at RT = 2.37 min). They corresponded to the protonated [M + H]^+^ molecules of artemisinin and 4′-bromoflavone, respectively. Statistical correlation analysis between these two LC-MS features and ^1^H-NMR buckets of the corresponding microfractions enabled the calculation of a NMR pseudospectrum correlated to each LC-MS feature. Highly correlated ^1^H-NMR chemical shifts (>0.8) with *m*/*z* = 283.1566 at RT = 2.18 min are displayed in Figure 2C and match with the signals of artemisinin. Correlation values were finally used to filter signals of fraction G9 to H10 (Figure 2D,E, Figures S1 and S2) from the sum of the ^1^H-NMR of those fractions (Figure 2B). The two filtered-spectra clearly matched those of the pure standards. Some of the chemical shifts were however missing or not well highlighted in the filtered spectra (δ_H_ (ppm) 6.05, 1.41 for artemisinin and δ_H_ (ppm) 6.98 for 4′-bromoflavone). This was related to other compounds exhibiting similar chemical shifts, leading to lower correlation values.

SHY has been shown to be thus efficient for the deconvolution of coeluting compounds and very useful to improve compound identification. Application of the correlation spectra to the sum of selected microfractions was more efficient than directly to the crude mixture NMR profile (Figure 2A).

### 2.2. Microfractionation Combined with Biological Profiling to Highlight Relevant Biologically Active Compounds

To evaluate if correlation analysis can be used in combination with microfractionation to highlight features corresponding to bioactive compounds, all fractions were submitted to a cell-based assay [34]. QR induction was evaluated at two different concentrations. The tested concentrations (0.8% and 0.08%) were defined as a proportion of the full well content used for biological evaluation [27]. These two proportions corresponded to 4 μg and 0.4 μg of the microfraction content used for the evaluation, respectively; considering that the 40 mg of AE were equivalently distributed in each of the 80 well of the plate (0.5 mg/well). In fact, the extract was not distributed in such a way, however when a compound is eluted its amounts increase and decrease in consecutive wells and this phenomenon can also be observed with similar variations of the measured biological activity. Enzymatic activity in this cell-based assay was normalised according to the amount of viable cells. Consequently an estimation of microfraction toxicity through cell viability measurement is also available. This can be used to highlight the presence of toxic compounds. Therefore, both information (QR induction and cell viability) were used as a strategy to discover non-toxic compounds with high QR induction potential.

#### 2.2.1. Targeted Deconvolution of Biologically Active Compounds Using LC-MS Data

In every microfraction LC-MS profiles, the peak area of the most intense ion was related to one compound mixed in the AE. As a preliminary approach, the peak area of the most intense molecular ion species of the mixed compounds (mostly [M + H]^+^ or [M − H]^−^) extracted by MZmine 2 [37] were evaluated in a targeted manner. This led to a very clean data matrix where every feature corresponded to a single compound.

To highlight bioactive compounds, both QR induction and cell viability results were correlated to these filtered MS features. The measured values were transformed to improve relevance of the QR induction and cell viability correlation. The QR induction parameter has an overall theoretical distribution between 0 and +∞, centred on 1. A value of 0 corresponds to a complete inhibition of the QR, an induction value of 1 is related to the absence of alteration of the activity of the enzyme and a value higher than 1 corresponds to an induction of its activity (however no real limit could be defined). Therefore, these values were log-transformed leading to values centred on 0 (*=log*(*1*)) with a distribution between –∞ (*=log*(*0*)) to +∞ (*=log*(*+∞*)). In addition, log-transformation of the data resulted in the conversion of multiplicative error into additive error, which is generally improving statistical evaluation of the data [38]. Similarly, the cell viability values were also transformed. The raw evaluation generates values from 0 (no cell growth) to 1 (normal cell growth), centred on 0.5. These values were transformed using the logit function to convert an “S”–shaped distribution into an approximately linear relationship with a range between –∞ (*=logit*(*0*)) and +∞ (*=logit*(*1*)), centred on 0 (*=logit*(*0.5*)) [39]. Such transformations should provide an improved relation between peak area, which are linearly linked to concentrations, and biological properties.

**Figure 3:**
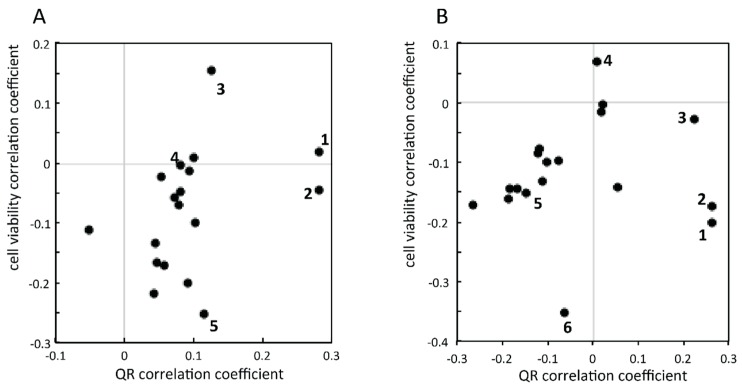
Correlation scatter plots between the two biological evaluations (x axis: QR induction; y axis: cell viability) with compound peak area in the different microfractions. The biological assays were evaluated at 0.08% (**A**) and at 0.8% (**B**). Raw values were transformed using log for QR induction and logit function for cell viability, respectively, before correlation calculation. Some compounds are highlighted in the figure: (**1**) 18-β-glycyrrhetinic acid; (**2**) 4′-bromoflavone; (**3**) artemisinin; (**4**) phenacetin; (**5**) taxifoline; (**6**) chelidonine.

As a consequence, all compounds were characterised by their correlation coefficient for both activities (at concentrations of 0.8% and 0.08%) and the data were separately displayed using a scatter plot (Figure 3). At 0.08% concentration (Figure 3A), two compounds were characterised by a high QR induction potential (*i.e.*, 18-β glycyrrhetinic acid and 4′-bromoflavone), revealed by a high correlation between their relative concentration within the microfractions and QR activity. Similar results were obtained at the higher concentration of 0.8% (Figure 3B). Complementarily, cell viability showed that some compounds (such as chelidonine or taxifoline) were characterised by high correlation values with toxicity (negative correlation with cell viability).

It is important to note that the separation between active molecules was improved at the lower concentration (0.08%) in Figure 3. This is highlighted by the example of artemisinin (**3**) in comparison to 4′-bromoflavone (**2**). On one hand, the artemisinin (**3**) correlation with QR induction is different between 0.8% and 0.08% (correlation value of 0.14 and 0.22, respectively). On the other hand, 4′-bromoflavone (**2**) has a similar correlation at both concentrations (correlation value of 0.26 and 0.28, at 0.8% and 0.08% respectively). This difference could be explained by exploring the data. Both compounds were coeluting; however, their chromatographic behaviour was not similar. When evaluated at high concentration (0.8%), a QR induction activity was detected in all fractions containing artemisinin (**3**) and 4′-bromoflavone (**2**). In the first fractions where artemisinin was detected, 4′-bromoflavone (**2**) could also be observed at low, but sufficient, concentration to reveal high QR induction. Therefore, the discrimination between those two compounds remained difficult using correlations. In contrast when evaluated at lower concentration (0.08%), the amount of 4’-bromoflavone (**2**) was 10 time lower in tested wells and thus the discrimination between highly active, inactive and slightly active microfractions was possible. Therefore, this demonstrated the importance of concentration in biological evaluation through correlation and clearly the low concentration measurement proved that only 4′-bromoflavone (**2**) was active. The evaluation at lower concentration allowed bioactive compounds to be highlighted more selectively. Having the possibility to compare bioactivity results at low and high concentrations provides important information to correctly assign the activity of a given compound in case of coelution. However, since concentration cannot be easily evaluated by MS responses in a real extract, this constitutes a limitation.

Finally, from the compounds mixed within the AE, two came out as potential QR inductors. 4′-Bromoflavone (**2**) was used in this study as a positive control as it is usually used in routine evaluation of compounds [40,41,42]. In addition, 18-β-glycyrrhetinic acid (**1**) was also revealed to possess QR induction potency and this constitutes a new finding.

#### 2.2.2. Untargeted Deconvolution of Biologically Active Compounds Using LC-MS Data

**Figure 4:**
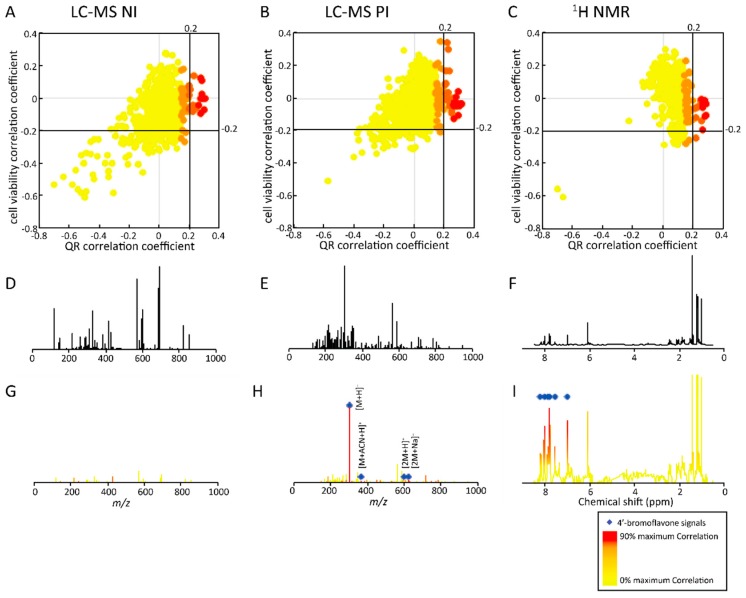
Correlation scatter plots between the two biological evaluations (x axis: QR induction—QR induction; y axis: cell viability) with features peak area in the different microfractions and the spectra corresponding to the active zone before and after activity filtering. Three untargeted approaches were evaluated using features detected by NI LC-MS (**A**); PI LC-MS (**B**) and ^1^H-NMR profiling (**C**); Correlations were calculated using log- and logit-transformed values from QR induction and cell viability assays, respectively. The sum spectrum of the active zone (B10-G10) are represented for the three analytical techniques (**D**–**F**); Based on correlation values, QR induction active features are highlighted (correlation value > 0.2) in the NI (**G**); PI (**H**); ^1^H-NMR (**I**).

In a second step, LC-MS fingerprints were evaluated in an unsupervised manner. Peak detection was performed automatically using MZmine 2 software and all extracted features were taken into consideration, in contrast to the targeted approach discussed above [37]. All detected peaks were correlated with both biological activities evaluated at 0.08% concentration using log- and logit- transformed data from QR and cell viability assays, respectively [34]. The results are represented in Figure 4A,B as correlation scatter plots. This approach allowed features responsible for high QR induction or low cell viability to be rapidly detected. NI and PI showed similar correlation patterns with many features without particular correlation with QR induction and cell viability (Figure 4A,B). Only few features were associated with low cell viability (correlation < −0.2) and QR inhibition (QR induction correlation < −0.2), as observed in the bottom left area of the correlation scatter plots in Figure 4A,B. These features are of no interest for natural product drug discovery because of their high toxicity combined with QR inhibition.

Focussing on the selection of the most relevant features (*i.e.*, high QR induction with low toxicity — no negative correlation with cell viability), only few features showed correlation with QR induction (correlation > 0.2). Similarly, a very small number of those specific features were characterised by a correlation with low cell viability < −0.2; those features were excluded as they exhibit correlation with measured toxicity. Therefore, only a limited number of features could be responsible for the cancer chemopreventive activity evaluated within this microfractionation experiments.

To identify the compounds of interest, selected features were searched within the sum NI and PI MS spectra of active microfractions (B10–G10) represented in Figure 4D,E. In the filtered NI spectrum (Figure 4G), only ions of low intensity were highlighted. In contrast, only main ions were evidenced in the PI spectra by this approach (Figure 4H). They corresponded to *m*/*z* = 300.9882, 342.0194, 600.9724, 622.9507. The comparison of those detected *m*/*z* could be related to different adducts of the same compound (MW = 301.13 g/mol) [M + H]^+^, [M + acetonitrile + H]^+^, [2M + H]^+^, [2M + Na]^+^, respectively [43]. All these features corresponded to 4'-bromoflavone. This observation was further confirmed by the isotopic pattern of the [M + H]^+^, [M + acetonitrile + H]^+^ adducts showing the presence of one bromine, and the isotopic pattern of the [2M + H]^+^, [2M + Na]^+^ adducts corresponding to the presence of two bromines. Similarly to the targeted approach, the untargeted approach based on the correlation of all detected MS features allowed the fast deconvolution of coeluting bioactive compounds in the microfractionation step. This proof of concept on the AE indicates that the approach is also applicable to the identification of bioactive compounds within a real crude extract where coelution problems are manifold during any fractionation step.

#### 2.2.3. Untargeted Deconvolution of Biologically Active Compounds Using ^1^H-NMR Data

Both targeted and untargeted LC-MS data mining strategies enabled the correct identification of features of the bioactive compounds (4′-bromoflavone). Each of the 80 microfractions were furthermore analysed by ^1^H-NMR to evaluate if a similar correlation experiment could unambiguously highlight characteristic NMR signals related to bioactive compounds. The area of each bucket over the microfractions was correlated with QR induction and cell viability using transformed biological data (log- and logit-transformed data for QR induction and cell viability evaluation, respectively) [34]. Figure 4C shows the correlation scatter plot from which the features of interest were selected. The correlation pattern generated using ^1^H-NMR (Figure 4C) was very similar to those observed using both NI and PI LC-MS data (Figure 4A,B). This highlighted the consecutive microfractions where the concentrations of all compounds varied (concentration increase followed by a decrease across a given LC microfraction peak). In addition, features with QR induction correlation > 0.2 corresponded to compounds exhibiting higher QR induction. Interestingly, this specific limit is the same as for the analysis of NI and PI LC-MS data.

To identify the compounds of interest with high QR induction correlations, highlighted ^1^H-NMR zones were selected (as this was the case for LC-MS) in the proton spectrum of the active microfractions (B10-G10—Figure 4F). This approach allowed the selection of specific NMR features (chemical shift and intensities) related to the bioactive compound (Figure 4I). As expected, these correlated NMR features highlighted perfectly matched those of the bioactive 4′-bromoflavone—δ_H_ (ppm) 8.18 (1H, d), 8.02 (2H, d), 7.87 (1H, t), 7.78 (1H + 2H, m), 7.53 (1H, t) and 6.98 (1H, s).

Thus both untargeted LC-MS and ^1^H-NMR correlation approaches were successful for identifying spectroscopic features correlated with bioactivity and enabled a satisfactory deconvolution and identification of the bioactive compound in this artificial mixture.

This approach is thus applicable to crude natural extracts and should provide an efficient means to identify and dereplicate bioactive compounds rapidly without the need for extensive and tedious purification steps, by the simple use of a single microfraction separation step.

## 3. Experimental Section

### 3.1. Creation of the Artificial Extract

The AE was created by mixing together the same mass of each of the following standard compounds: l-epicatechin, chelidonine, papaverine, luteolin, phenacetin, 1,8-dihydroxyanthraquinone, 18-β-glycyrrhetinic acid, quinidine, resorcinol, artemisinin, dihydroquercetin (taxifolin), boldine, naringenin, 5,8-dihydroxy-1,4-naphtoquinone (naphthazarin), khellin, aloin, xanthotoxin and 4'-bromoflavone (positive bioactive standard).

### 3.2. Semi-Preparative Microfractionation of the Synthetic Extract by Reversed Phase HPLC

The synthetic extract was microfractionated using a semi-preparative HPLC-UV system (Spot Prep, Armen, Saint-Avé, France) using a C_18_ column (XBridge Prep C18, 5 µm, 10 × 250 mm, Waters, Milford, MA, USA). The procedure was developed at the analytical scale and geometrically transferred to the semi-preparative scale using the HPLC calculator 3.0 [35,36]. The flow rate was set at 4.7 mL/min and 40 mg of synthetic extract dissolved in 3 mL of methanol were injected. Chromatographic separation was achieved using a solvent system composed of (A) H_2_O and (B) acetonitrile. The microfractionation procedure consisted of four consecutive gradients: from 24%B to 40.3%B in 16 min, from 40.3%B to 48%B in 5 min, from 48%B to 92%B in 1 min and finally from 92%B to 95%B in 2 min. The column was then washed by 20 min at 95%B. Microfractions were collected every 0.33 min and yielded 80 fractions that were transferred to a 96-deepwell plate and dried by a centrifugal evaporator (Genevac, Suffolk, UK).

### 3.3. UHPLC-TOFMS Profiling of the Microfractions

UHPLC-TOFMS analyses were performed using a Waters Micromass-LCT Premier Time-of-Flight mass spectrometer equipped with an ESI interface coupled to an Acquity UPLC system (Waters). Detection was achieved using both the PI and NI mode sequentially in one analysis. The *m*/*z* range was set to 100–1000 in centroid mode with a scan time of 0.25 s and an inter-scan delay of 0.01 s. The ESI conditions in the PI and NI modes were as follows: capillary voltage of 2800 V, cone voltage of 40 V, source temperature of 120 °C, desolvation temperature of 250 °C, cone-gas flow of 20 L/h, and desolvation-gas flow 600 L/h. For internal calibration, a 5 μg/mL solution of leucine-enkephalin (Sigma-Aldrich, St. Louis, MO, USA) was infused through the lock-mass probe at a flow rate of 5 μL/min using a second LC-10ADvp LC pump (Shimadzu, Kyoto, Japan).

UHPLC-TOFMS fingerprints were recorded with a 50 × 1 mm i.d., 1.7 μm Acquity BEH C_18_ UPLC column (Waters) in gradient mode at a flow rate of 0.3 mL/min with the following solvent system: (A) 0.1% *v*/*v* formic acid (FA) in water (ULC/MS-grade Biosolve, Valkenswaard, The Netherlands); (B) 0.1% *v*/*v* FA in acetonitrile(ULC/MS-grade Biosolve). The gradient was increased from 5%B to 95%B in 4 min. The column was then washed for 0.8 min with 95%B, reconditioned to 5%B in 0.1 min and finally equilibrated with 5%B in 1.1 min. The total analysis time including re-equilibration of the column was thus 6 min providing a complete analysis of all fractions in approximately 8 h. The temperature was maintained at 40 °C, and the injection volume was 1 μL of an approximately 0.5 mg/mL (considering that the whole AE was equally divided in the 80 microfractions). The analyses were performed randomly and included blank samples after every 10 sample runs. The sample list was randomly generated using a dedicated Excel macro [44].

### 3.4. Targeted and Untargeted Peak Picking of LC-MS Data

Native MassLynx data (Waters) were converted into netCDF (common data format) using DataBridge software (Waters). Automatic feature detection was performed between 0.4 and 4.5 min with MZmine 2 software [37] using parameters selected according to the TOFMS detector. For targeted approach *m*/*z* tolerance, noise level and retention time tolerances were set to 40 ppm, 0 count and 0.3 min, respectively. For untargeted approaches the classical steps were used. Peaks with a width of at least 0.03 s and an intensity greater than 100 counts (both NI and PI) were picked with a 20 ppm *m*/*z* tolerance and the generated peak lists were deconvoluted. Deisotope filtering was applied using the “isotopic peaks grouper” module with tolerance parameters adjusted to 0.03 s and 20 ppm. Feature alignment and gap filling were achieved with a *m*/*z* tolerance of 40 ppm and a RT tolerance of 0.33 min. Features detected in blank samples were removed.

### 3.5. ^1^H-NMR Profiling of the Microfractions and NMR Data Matrix Creation

The content of each well was dissolved in 200 µL CD_3_OD. All wells were transferred into 96 well plate filter (Waters) and filtered to a 96 deep well plate by centrifugation (Genevac, Suffolk, UK). The final plate containing the dissolved microfractions was sealed prior to analysis. Each sample was analysed by Microflow NMR on a Unity Inova 500 MHz NMR instrument (Varian, Palo Alto, CA, USA) equipped with a 5 µL microflow NMR probe from Protasis/MRM (Savoy, IL, USA) with an active volume of 1.5 µL. An automatic careful cleaning was achieved after each microfraction analysis to avoid sample cross-contamination. Bucketing of the ^1^H-NMR data was performed with a bucket width of 0.02 ppm between 0.5 and 8.5 ppm using MestreNova (Mestrelab Research S.L., Santiago de Compostela, Spain). Specific regions covering solvent peaks (CD_3_OD—3.36−3.26 ppm—, and residual HDO—5.00−4.56 ppm) were excluded from the data matrix. In total, 374 buckets remained after data preprocessing.

### 3.6. Quinone Reductase Induction Activity

Hepa1c1c7 cells (ATCC, Rockville, MD, USA) were cultured according to the ATCC recommendations in α-modified Minimal Essential Medium (Life Technologies, Zug, Switzerland) containing 2 mM glutamine, supplemented with 100 units/mL penicillin, 100 μg/mL streptomycin, and 10% foetal calf serum at 37 °C, 5% CO_2_, and humidified atmosphere. The method described by Kang and Pezzuto [45] was used. In brief, QR activity was determined in a 96-well format after two days sample incubation. Cells were then lysed and the NADPH-dependent menadiol-mediated reduction of 3-(4,5-dimethylthiazo-2-yl)-2,5-diphenyltetrazolium bromide (MTT) to the corresponding blue fromazan was measured on a PowerWave HT microplate spectrophotometer (BioTek Instruments, Luzern, Switzerland) at 595 nm. In parallel, the amount of viable cells was determined by protein quantification using crystal violet staining and measurement of the absorption at 595 nm on the aforementioned spectrophotometer. Microfractions were tested at a fixed concentration of 0.08% and 0.8% collection well content. The full microfractionation plate was tested in duplicate.

### 3.7. Statistical Correlation

All calculations were performed using Excel (Microsoft, Redmond, WA, USA) as standard software. When mentioned, QR induction data were log-transformed and cell viability data were logit-transformed (Equation (1)). None of the LC-MS or ^1^H-NMR data were normalized or transformed prior to the statistical analysis. Pearson correlations were calculated using the “=CORREL(array1, array2)” function from Excel between each features detected in each microfraction and the corresponding biological evaluation (array1 corresponding to the intensity values of one feature and array2 corresponding to the corrected bioassay value; both array need to be of the same length):

(1) $$Logit\left(x\right)=ln\left(\frac{x}{1-x}\right)$$

## 4. Conclusions

A deconvolution method was devised and tested in a mixture of natural products (artificial extract, AE) to overcome problems of bioactive compound identification in microfractions when coelution occurs with other matrix constituents. The approach demonstrated that the differences in amounts among microfractions induced by slight changes in chromatographic behaviours, LC-MS and NMR data can be correlated through SHY. This led to clean NMR spectra of the detected LC-MS features in case of coelution.

For at-line HPLC coupling, bioactivity values of QR induction and cell viability values were appropriately transformed to improve correlations. Correlation between QR induction and both LC-MS data and ^1^H-NMR data clearly highlighted the positive control used in this study, *i.e.*, 4′-bromoflavone. Therefore this improved microfractionation-guided approach led to an efficient selection of the features of interest, despite the presence of hiding inactive constituents coeluting with highly active natural product. In addition, the correlation between the LC-MS and ^1^H-NMR data led to improved selection of NMR signals corresponding to a specific MS feature, in the context of dereplication.

The application of correlation experiments in conjunction with rapid fractionation protocol represent thus an interesting approach for a rapid deconvolution of mixtures of NPs without the need for a complete and tedious purification of bioactive constituents. The results obtained on AE indicated that even higher throughput microfractionation might be used and still enable the deconvolution of coeluting compounds. This approach should now be applied for the efficient dereplication of QR inducers within crude natural extracts where a high number of constituents occurs over a large range of concentrations.

## Supplementary Materials

The followings are available online at http://www.mdpi.com/1420-3049/21/3/259/s1. Figure S1 and S2: Example of statistical deconvolution on the coelution of artemisinin and 4′-bromoflavone.

## References

[1] Newman D.J., Cragg G.M. (2012). Natural products as sources of new drugs over the 30 years from 1981 to 2010. J. Nat. Prod..

[2] Rosén J., Gottfries J., Muresan S., Backlund A., Oprea T.I. (2009). Novel chemical space exploration via natural products. J. Med. Chem..

[3] Zhu F., Qin C., Tao L., Liu X., Shi Z., Ma X., Jia J., Tan Y., Cui C., Lin J. (2011). Clustered patterns of species origins of nature-derived drugs and clues for future bioprospecting. Proc. Natl. Acad. Sci. USA.

[4] David B., Wolfender J.-L., Dias D.A. (2014). The pharmaceutical industry and natural products: Historical status and new trends. Phytochem. Rev..

[5] Koehn F.E., Carter G.T. (2005). The evolving role of natural products in drug discovery. Nat. Rev. Drug Discov..

[6] Lam K.S. (2007). New aspects of natural products in drug discovery. Trends Microbiol..

[7] Gonzalez-Sabin J. (2012). Natural products: Back to the future in drug discovery. Biochem. Pharmacol..

[8] Roemer T., Xu D., Singh S.B., Parish C.A., Harris G., Wang H., Davies J.E., Bills G.F. (2011). Confronting the challenges of natural product-based antifungal discovery. Chem. Biol..

[9] Bertrand S., Schumpp O., Bohni N., Monod M., Gindro K., Wolfender J.-L. (2013). *De novo* production of metabolites by fungal co-culture of *Trichophyton rubrum* and *Bionectria ochroleuca*. J. Nat. Prod..

[10] Glauser G., Guillarme D., Grata E., Boccard J., Thiocone A., Carrupt P.-A., Veuthey J.-L., Rudaz S., Wolfender J.-L. (2008). Optimized liquid chromatography-mass spectrometry approach for the isolation of minor stress biomarkers in plant extracts and their identification by capillary nuclear magnetic resonance. J. Chromatogr. A.

[11] Potterat O., Hamburger M. (2014). Combined use of extract libraries and HPLC-based activity profiling for lead discovery: potential, challenges, and practical considerations. Planta Med..

[12] Potterat O., Hamburger M. (2013). Concepts and technologies for tracking bioactive compounds in natural product extracts: Generation of libraries, and hyphenation of analytical processes with bioassays. Nat. Prod. Rep..

[13] Kool J., Giera M., Irth H., Niessen W.A. (2011). Advances in mass spectrometry-based post-column bioaffinity profiling of mixtures. Anal. Bioanal. Chem..

[14] Simões-Pires C.A., Queiroz E.F., Henriques A.T., Hostettmann K. (2005). Isolation and on-line identification of anti-oxidant compounds from three *Baccharis* species by HPLC-UV-MS/MS with post-column derivatisation. Phytochem. Anal..

[15] Rhee I.K., Appels N., Luijendijk T., Irth H., Verpoorte R. (2003). Determining acetylcholinesterase inhibitory activity in plant extracts using a fluorimetric flow assay. Phytochem. Anal..

[16] De Boer A.R., Alcaide-Hidalgo J.M., Krabbe J.G., Kolkman J., van Emde Boas C.N., Niessen W.M.A., Lingeman H., Irth H. (2005). High-temperature liquid chromatography coupled on-line to a continuous-flow biochemical screening assay with electrospray ionization mass spectrometric detection. Anal. Chem..

[17] Wubshet S.G., Nyberg N.T., Tejesvi M.V., Pirttilä A.M., Kajula M., Mattila S., Staerk D. (2013). Targeting high-performance liquid chromatography-high-resolution mass spectrometry-solid-phase extraction−nuclear magnetic resonance analysis with high-resolution radical scavenging profiles-bioactive secondary metabolites from the endophytic fungus *Penicillium namyslowskii*. J. Chromatogr. A.

[18] Wolfender J.-L., Marti G., Thomas A., Bertrand S. (2015). Current approaches and challenges for the metabolite profiling of complex natural extracts. J. Chromatogr. A.

[19] Bohni N., Cordero-Maldonado M.L., Maes J., Siverio-Mota D., Marcourt L., Munck S., Kamuhabwa A.R., Moshi M.J., Esguerra C.V., de Witte P.A.M. (2013). Integration of microfractionation, QNMR and zebrafish screening for the *in vivo* bioassay-guided isolation and quantitative bioactivity analysis of natural products. PLoS ONE.

[20] Challal S., Buenafe O.E.M., Queiroz E.F., Maljevic S., Marcourt L., Bock M., Kloeti W., Dayrit F.M., Harvey A.L., Lerche H. (2014). Zebrafish-bioassay guided microfractionation identifies anticonvulsant steroid glycosides from the Philippine medicinal plant *Solanum torvum*. ACS Chem. Neurosci..

[21] El-Elimat T., Figueroa M., Ehrmann B.M., Cech N.B., Pearce C.J., Oberlies N.H. (2013). High-resolution MS, MS/MS, and UV database of fungal secondary metabolites as a dereplication protocol for bioactive natural products. J. Nat. Prod..

[22] Smyth W.F., Smyth T.J.P., Ramachandran V.N., O’Donnell F., Brooks P. (2012). Dereplication of phytochemicals in plants by LC-ESI-MS and ESI-MS^n^. Trends Anal. Chem..

[23] Grosso C., Jäger A.K., Staerk D. (2012). Coupling of a high-resolution monoamine oxidase-A inhibitor assay and HPLC–SPE–NMR for advanced bioactivity profiling of plant extracts. Phytochem. Anal..

[24] Queiroz E.F., Wolfender J.L., Atindehou K.K., Traore D., Hostettmann K. (2002). On-line identification of the antifungal constituents of *Erythrina vogelii* by liquid chromatography with tandem mass spectrometry, ultraviolet absorbance detection and nuclear magnetic resonance spectrometry combined with liquid chromatographic micro-fractionation. J. Chromatogr. A.

[25] Yuliana N., Jahangir M., Verpoorte R., Choi Y. (2013). Metabolomics for the rapid dereplication of bioactive compounds from natural sources. Phytochem. Rev..

[26] Bertrand S., Petit C., Marcourt L., Ho R., Gindro K., Monod M., Wolfender J.-L. (2014). HPLC profiling with at-line microdilution assay for the early identification of antifungal compounds in plants from French Polynesia. Phytochem. Anal..

[27] Landreau A., Bertrand S., Simoes-Pires C., Marcourt L., dan Bach T., Litaudon M., Guilet D., Richomme P., Carrupt P.-A., Wolfender J.-L. (2016). Guttiferones as acetylcholinesterase inhibitors retrieved by normal phase HPLC profiling of apolar plant extract. Nat. Prod. Res..

[28] Crockford D.J., Holmes E., Lindon J.C., Plumb R.S., Zirah S., Bruce S.J., Rainville P., Stumpf C.L., Nicholson J.K. (2006). Statistical heterospectroscopy, an approach to the integrated analysis of NMR and UPLC-MS data sets: Application in metabonomic toxicology studies. Anal. Chem..

[29] Crockford D.J., Maher A.D., Ahmadi K.R., Barrett A., Plumb R.S., Wilson I.D., Nicholson J.K. (2008). ^1^H-NMR and UPLC-MS(E) statistical heterospectroscopy: Characterization of drug metabolites (xenometabolome) in epidemiological studies. Anal. Chem..

[30] Marti G., Boccard J., Mehl F., Debrus B., Marcourt L., Merle P., Delort E., Baroux L., Sommer H., Rudaz S. (2014). Comprehensive profiling and marker identification in non-volatile citrus oil residues by mass spectrometry and nuclear magnetic resonance. Food Chem..

[31] Yuliana N., Khatib A., Choi Y., Verpoorte R. (2011). Metabolomics for bioactivity assessment of natural products. Phytother. Res..

[32] Wiegandt A., Meyer B. (2014). Unambiguous characterization of *N*-glycans of monoclonal antibody cetuximab by integration of LC-MS/MS and ^1^H-NMR Spectroscopy. Anal. Chem..

[33] Behnken H., Fellenberg M., Koetzler M., Jirmann R., Nagel T., Meyer B. (2012). Resolving the problem of chromatographic overlap by 3D cross correlation (3DCC) processing of LC, MS and NMR data for characterization of complex glycan mixtures. Anal. Bioanal. Chem..

[34] Inui T., Wang Y., Pro S.M., Franzblau S.G., Pauli G.F. (2012). Unbiased evaluation of bioactive secondary metabolites in complex matrices. Fitoterapia.

[35] Guillarme D., Nguyen D.T.T., Rudaz S., Veuthey J.-L. HPLC Calculator 3.0. http://www.unige.ch/sciences/pharm/fanal/lcap/telechargement.htm.

[36] Guillarme D., Nguyen D.T.T., Rudaz S., Veuthey J.-L. (2008). Method transfer for fast liquid chromatography in pharmaceutical analysis: Application to short columns packed with small particle. Part II: Gradient experiments. Eur. J. Pharm. Biopharm..

[37] Pluskal T., Castillo S., Villar-Briones A., Oresic M. (2010). MZmine 2: Modular framework for processing, visualizing, and analyzing mass spectrometry-based molecular profile data. BMC Bioinf..

[38] Van den Berg R.A., Hoefsloot H.C., Westerhuis J.A., Smilde A.K., van der Werf M.J. (2006). Centering, scaling, and transformations: Improving the biological information content of metabolomics data. BMC Genom..

[39] Bewick V., Cheek L., Ball J. (2005). Statistics Review 14: Logistic regression. Crit. Care.

[40] Al-Massarani S.M., Bertrand S., Nievergelt A., Mohammed El-Shafae A., Abdullah Al-Howiriny T., Musayeib Al-Musayeib N., Cuendet M., Wolfender J.-L. (2012). Acylated pregnane glycosides from *Caralluma sinaica*. Phytochemistry.

[41] Giacomelli E., Bertrand S., Nievergelt A., Zwick V., Simoes-Pires C., Marcourt L., Rivara-Minten E., Cuendet M., Bisio A., Wolfender J.-L. (2013). Cancer chemopreventive diterpenes from *Salvia corrugata*. Phytochemistry.

[42] Misico R.I., Song L.L., Veleiro A.S., Cirigliano A.M., Tettamanzi M.C., Burton G., Bonetto G.M., Nicotra V.E., Silva G.L., Gil R.R. (2002). Induction of quinone reductase by withanolides. J. Nat. Prod..

[43] Kind T., Fiehn O. (2007). Seven golden rules for heuristic filtering of molecular formulas obtained by accurate mass spectrometry. BMC Bioinf..

[44] Bertrand S., Schumpp O., Bohni N., Bujard A., Azzollini A., Monod M., Gindro K., Wolfender J.-L. (2013). Detection of metabolite induction in fungal co-cultures on solid media by high-throughput differential ultra-high pressure liquid chromatography-time-of-flight mass spectrometry fingerprinting. J. Chromatogr. A.

[45] Kang Y.-H., Pezzuto J.M. (2004). Induction of quinone reductase as a primary screen for natural product anticarcinogens. Methods Enzymol..

