# Supplementary material for: Statistical Correlations between HPLC Activity-Based Profiling Results and NMR/MS Microfraction Data to Deconvolute Bioactive Compounds in Mixtures
Source: Molecules. 2016 Feb 24;21(3):259. doi: 10.3390/molecules21030259 (PMC6274519; doi:10.3390/molecules21030259)
Supplement: Supplementary file 1 [file molecules-21-00259-s001.pdf]

Supplementary Materials: Statistical Correlations between HPLC Activity-Based Profiling Results and NMR/MS Microfractions Data to Deconvolute Bioactive Compounds in Mixture

Samuel Bertrand, Antonio Azzollini, Andreas Nievergelt, Julien Boccard, Serge Rudaz, Muriel Cuendet and Jean-Luc Wolfender

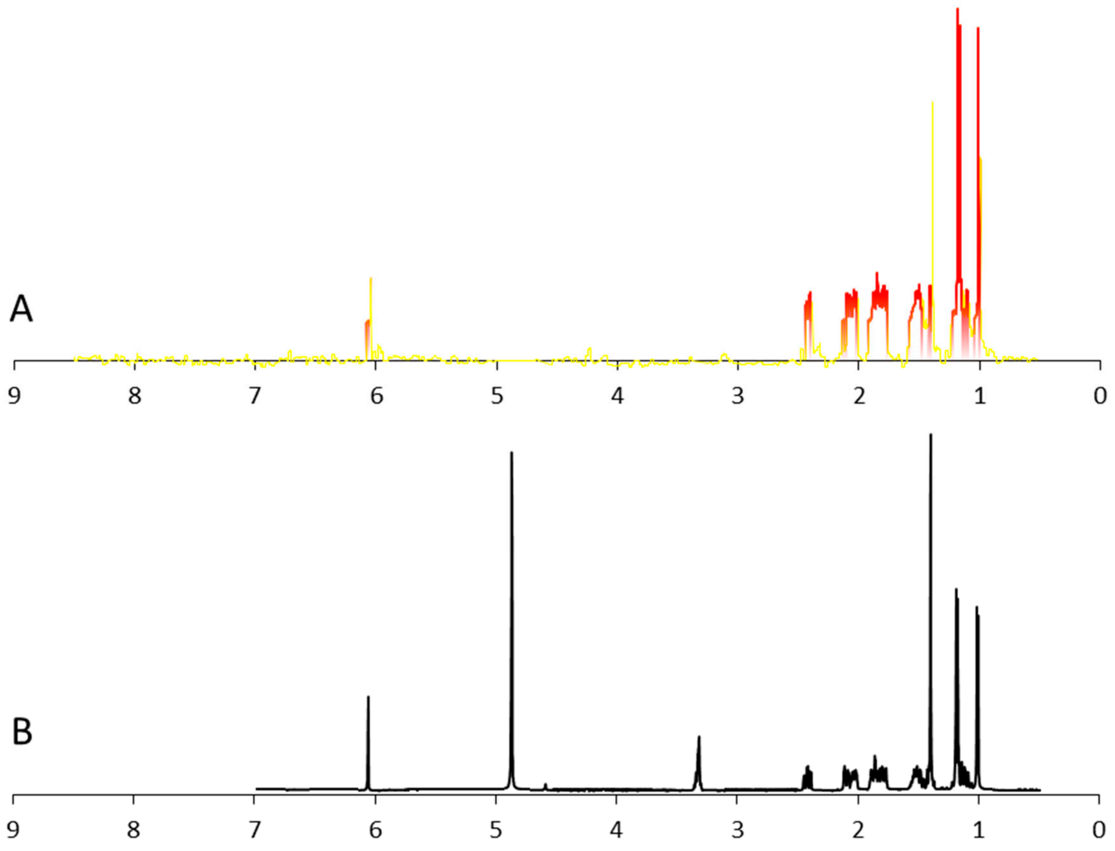

Figure S1. Example of statistical deconvolution on the coelution of artemisinin and 4′-bromoflavone. (**A**) filtered ^{1}H-NMR pseudospectrum corresponding to feature RT = 2.18 min and m/z = 283.1566 (PI) – artemisinin as $[M + H]^{+}$ adduct; (**B**) real spectrum of artemisinin in deuterated methanol.

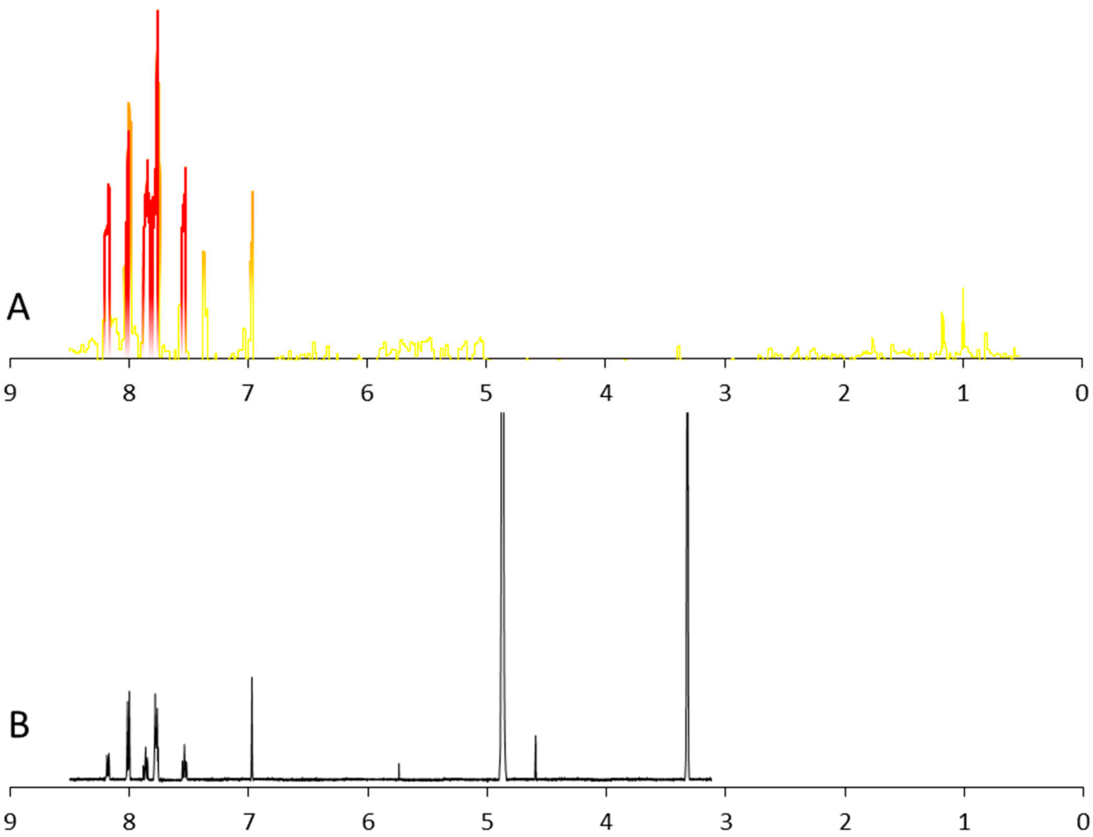

Figure S2. Example of statistical deconvolution on the coelution of artemisinin and 4′-bromoflavone. (**A**) filtered ^{1}H-NMR pseudospectrum corresponding to feature RT = 2.37 min and *m/z* = 300.9882 (PI)—4′-bromoflavone as $[M + H]^+$ adduct; (**B**) real spectrum of 4′-bromoflavone in deuterated methanol.
